# Trends in Childhood Cancer in Kuwait: Data From the 2004-2017 Registry

**DOI:** 10.7759/cureus.13333

**Published:** 2021-02-13

**Authors:** Maha J Bourusly, Muna H Burahma, Nisreen Khalifa, Hubert Motti, Sahar Kaleefa, Mohammad Adil, Suad Alanzi, Medhat Alshazli

**Affiliations:** 1 Pediatric Hematology and Oncology, National Bank of Kuwait Specialized Hospital for Children, Sabah, KWT; 2 Pediatrics, National Bank of Kuwait Specialized Hospital for Children, Sabah, KWT; 3 Pediatric Oncology, National Bank of Kuwait Specialized Hospital for Children, Sabah, KWT; 4 Pediatric Oncology, Ministry of Health, Kuwait City, KWT

**Keywords:** cancer registry, neoplasm, leukemia, childhood cancer, pediatric oncology

## Abstract

Background and objective

There is a lack of updated data regarding pediatric cancer in Kuwait, with no separate childhood cancer registry available in the country prior to this study. We aimed to determine the trends in different cancer types with respect to international statistics, in order to understand their nature and identify gaps in their diagnosis and management.

Methods

This retrospective study was based on data obtained from the first national childhood cancer registry of the National Bank of Kuwait (NBK) Specialized Hospital for Children, the only hospital that manages pediatric cancer patients in Kuwait. The registry included the data of all children with cancer in Kuwait from 2004 to 2017 and had complete data files.

Findings

The total number of patients in the childhood cancer registry was 1,387. A total of 1,009 files met our eligibility criteria. In 2017, the incidence of childhood leukemia was determined to be six per 100,000 people, and for other cancers, it was 12.6 per 100,000 people. The most common cancer was leukemia (457 patients), followed by lymphoma (141 patients), and brain tumors (92 patients). The majority of children received chemotherapy (n=891, 88.3%). Post induction or treatment, the majority of patients achieved complete remission (n=790, 78.3%). The overall survival rate of children with cancer was around 80%. Major complications developed in 9% of patients.

Conclusion

These findings highlight the importance of hospital‐based cancer registries. Active data management programs are essential to monitor outcomes, measure the effectiveness of current practice, and improve the quality of care.

## Introduction

Globally, more than 250,000 children are diagnosed with cancer each year. The incidence of pediatric malignancy has been on the rise for many reasons, including the improvement in the diagnosis and documentation of cancer patients [1]. According to the Central Statistical Bureau Annual Bulletin of Vital Statistics for Birth and Deaths, cancer was the second leading cause of death in adults in Kuwait between the years 2015-2017. Unfortunately, there are no specific mortality statistics for children below 14 years in Kuwait [2]. A published study based on the data of the Kuwaiti Cancer Registry (KCR) at the Kuwait Cancer Control Center (KCCCH) has reported that from 2010 to 2013, the highest five-year survival rate among patients aged more than 15 years old was in patients with lymphoma (96.3%, 95% CI: 91.4-100.0%) followed by acute lymphocytic leukemia (ALL) (88.4%, 95% Cl: 80.6-96.2%). However, between 2000 and 2013, the largest improvement in the survival rates of children was recorded among ALL patients, who showed a 12.3% increase in survival versus a 6.3% increase in lymphoma patients [3].

One of the main goals of the State of Kuwait 2035 Vision is the advancement of Kuwaiti healthcare [4]. Having a formal registry and documenting disease epidemiology and statistics play an important role in the planning and implementation of health plans and developing the public healthcare system [5]. Unfortunately, there is a lack of data and statistics on pediatric cancer in Kuwait, and data related to children with cancer are included in the adult national cancer registry. The objective of this study was to determine the trends and statistics related to different cancers affecting children in Kuwait.

## Materials and methods

Study design and data collection

This retrospective chart review used the data obtained from the national childhood cancer registry of the National Bank of Kuwait (NBK) Specialized Hospital for Children. All pediatric cancer patients in Kuwait are referred to this hospital and treated there. Data collection for the cancer registry started on May 12, 2016, and went on till December 2017, and included data of all children with cancer in Kuwait from January 2004 until December 2017. The datasheet used in the registry was finalized in accordance with the International Agency for Research on Cancer (IARC) guidelines and contained data regarding patient biography, investigations, clinical course, complications, and outcomes. The final data sheet was conceived after running a pilot study and reviewing registries from other countries and the Kuwait registry center. All children with cancer who attended the NBK Hospital and had complete data files were included in the study. Any patients with benign tumors or incomplete data files were excluded.

Entries were categorized according to the latest International Classification of Diseases (ICD), excluding stratification. The risk stratification of patients was based on the randomized United Kingdom National Acute Lymphoblastic Leukaemia (UKALL) 2003 study [6]. Investigators collected the data manually from the files of leukemia patients receiving treatment at the NBK Hospital from the year 2004 and from the files of patients with other cancers after 2005. Files from 2004 and 2005 belonged to leukemia patients only as other cancers were treated in adult hospitals prior to 2010, and hence their files were not available. After 2010, all children with cancer were treated at the NBK Hospital. Data from later dates (from 2010) were collected from electronic records, which were not available for earlier dates.

Data analysis

We analyzed the data collected to document the trends in childhood cancer in Kuwait. Data were processed and analyzed using SPSS Statistics for Windows, version 22 (IBM, Armonk, NY). Statistical analysis included quantitative descriptive analysis and summary statistics describing the frequency of diagnostic groups and subgroups of childhood cancer, in addition to survival data using the Kaplan-Meier survival curve.

Study population

Our study population included all children diagnosed with cancer in the state of Kuwait from 2004 to 2017. The details relating to the age of patients we studied were as follows: children under the age of 16 for the year 2017, children under the age of 15 from 2012 to 2017, and children under the age of 14 from 2004 to 2011. The difference in age range was due to the difference in the age of acceptance of patients at the NBK Hospital. Any child with an initial diagnosis of cancer in the state of Kuwait, regardless of nationality, is referred to the NBK Hospital for disease confirmation and treatment. Therefore, the cases included in this study represent the total number of childhood cancer patients in Kuwait, which amounted to a total of 1,387. After excluding duplicate files (n=125) and files with insufficient data (n=46), we analyzed the data from the remaining 1,009 files. Follow-up data were collected from the eligible records if they were available. The patients were followed up according to the routine practice followed at the NBK Hospital. Ethical approval was granted by the Standing Committee for Coordination of Health and Medical Research, and the Pediatric Departments Council at Kuwait’s Ministry of Health; the need for informed consent was waived as the data used was secondary data, with no exposure of patient identity. The research protocol was conducted according to the principles of the Declaration of Helsinki.

## Results

Out of 1,009 patient files, 457 were leukemia patients, and 552 were children with other malignancies. By reviewing the population details in the Kuwait census in the last two decades, we calculated the incidence rate for childhood leukemia as approximately six per 100,000 and for other cancers as nine per 100,000 for children below the age of 14 in 2011. Table 1 shows the number of childhood cancer patients presenting to the NBK Hospital per year and the number of files retrieved. In 2017, the incidence rate of childhood leukemia was calculated as six per 100,000, and for other cancers as 12.6 per 100,000. However, the variability in incidence should be considered with caution because the population of Kuwait obtained from the national statistics center was for ages 0-14 years, and our study population included children aged 0-16 years. Moreover, we used the 2018 census to calculate the incidence rate, and so there may be a discrepancy between the actual rates and the rates we calculated.

**Table 1:** Number of childhood cancer patients presenting to the NBK Hospital per year and the number of files retrieved NBK: National Bank of Kuwait

Year	New oncology patients	Files retrieved	New leukemia patients	Files retrieved	Total retrieved
2004	34	1	36	23	24
2005	53	12	31	21	33
2006	46	20	34	17	37
2007	59	27	29	22	49
2008	61	41	63	49	90
2009	62	37	34	28	65
2010	61	52	29	27	79
2011	64	54	46	46	100
2012	72	62	41	40	102
2013	52	43	34	33	76
2014	67	51	41	41	92
2015	74	67	31	28	95
2016	68	64	43	39	103
2017	77	66	45	44	110

Patient characteristics

**Figure 1:**
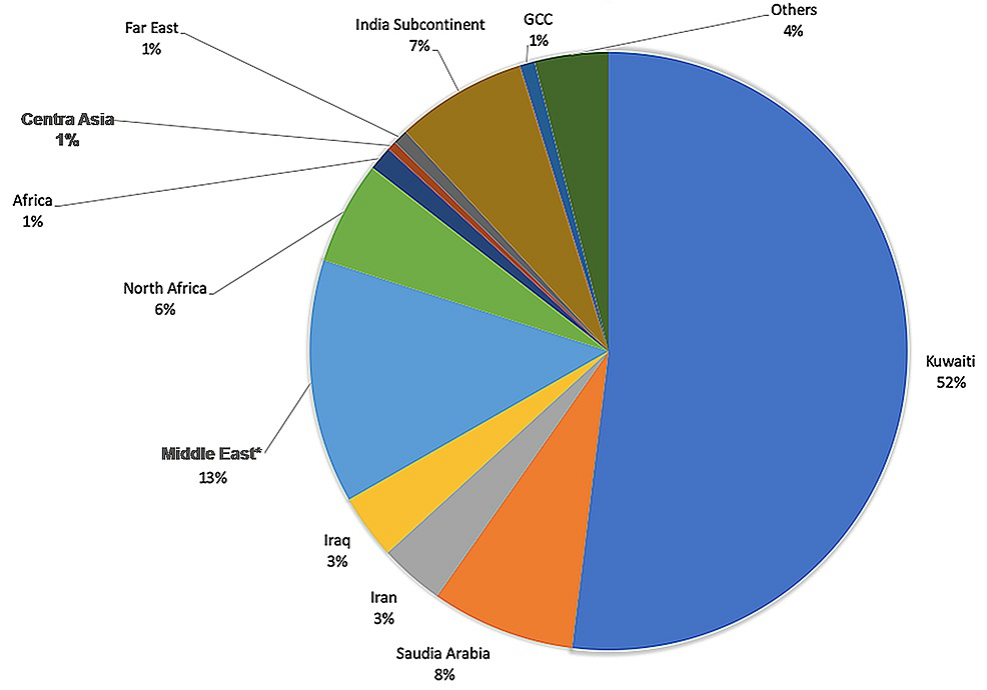
Nationalities of children with cancer presenting to the NBK Hospital in Kuwait between 2004-2017 *Middle East: all middle eastern countries excluding Saudi Arabia, Iran, Iraq, and Kuwait NBK: National Bank of Kuwait; GCC: Gulf Cooperation Council

There were 588 males and 421 females, with ages ranging from one month to 16 years; the mean age was six years. There were 593 Kuwaitis and 416 non-Kuwaitis of 28 other nationalities (Figure 1). Only 7% (70/939) of the patients had known genetic diseases and the most frequent genetic disease was Down syndrome (n=37, 3.7% of all population), followed by hereditary retinoblastoma (n=12, 2% of the population). Previous medical history was reported in 92 children, such as diabetes mellitus, cardiac diseases, central nervous system (CNS) diseases, and others. The most frequent medical condition was cardiac diseases (n=27) followed by CNS diseases (n=18) and respiratory diseases (n=15).

A positive family history of cancer was recorded in 298 files, either in a first-degree relative (n=33, 3.3%), second-degree relative (n=110, 10.9%), or an extended family member (n=155, 15.4%). The level of education of parents was assessed; 35.7% (n=357) of the parents were educated to the level of high school. The majority of parents had intermediate education (no high school certification) (n=552, 54.7%); in a few families, both parents did not receive any formal education (n=12, 1.2%), and data were missing in 88 files. The number of siblings was mentioned in 619 files (mean: 2.86; range: 0-22). Almost half of the patients were in preschool (49%, n=479), and 46.8% were at school, in one of the three stages of school education (n=470). Unfortunately, 4.2% (n=42) of children were school-aged but did not receive any formal education.

Diagnosis

Positive histology samples were used for diagnosis in 48.7% of patients (n=491), and positive laboratory tests or markers were used for diagnosis in 46.1% of patients (n=465). Immune phenotyping was frequently used as a diagnostic tool for leukemia but rarely used in other cancers. The most frequently recurrent immune-phenotyping values were CD10, CD19, CD45, CD34, and HLA-DR.

Cytogenetics were used for the diagnosis and prediction of the prognosis of childhood leukemia. Normal karyotypes with no aberrations were detected in 120 children, while 57 children showed normal karyotypes with detected aberrations. Ninety-nine children showed hyperdiploidy; while five children had hypoploidy. Cytogenetic analysis was not recorded in 597 patients (mainly in children with cancers other than leukemia).

Cancer types and disease risk

For the sake of convenience, we divided cancers into two categories: solid tumors (including brain tumors, lymphomas, and bone, renal, and other tumors), and leukemia. The main primary sites of cancer in non-leukemia patients were the abdomen and pelvis (34%) followed by the head and neck (33%). In the solid tumor group, 50.7% of patients (n=280) had localized tumors, 16% (n=92) had distant metastasis, and 16.1% (n=89) had localized tumors with direct extension.

The most prevalent cancer was leukemia, followed by lymphoma and brain tumors. The most common brain tumor in our registry was glioma (52.7%), followed by medulloblastoma (30.7%), anaplastic ependymoma (8.7%), and brain and spine tumors (6.5%). The most prevalent leukemia was ALL (80%), followed by acute myeloblastic leukemia (AML, 16.6%) and chronic myeloid leukemia (CML, 3%). Children with Down syndrome developed leukemia (n=35) and other solid tumors (n=2). Fourteen children with Down syndrome had AML, and 21 had ALL.

Risk stratification was performed, and patients were divided according to their risk into high-risk (30.8%, n=311), intermediate-risk (33%, n=333), and low-risk (29.1%, n=294) categories. There were 71 children with undetermined risk, or where the calculation of risk was non-applicable. The disease risk changed in certain children, mostly in children with leukemia for many reasons; either due to minimal residual disease (MRD) results (n=61), end of induction bone marrow results (n=12), or due to non-specific reasons (n=5).

Treatment and complications

Surgical procedures were performed for 510 patients; 100 patients underwent a second surgical procedure, and 13 underwent a third surgical procedure (Table 2). Post-surgical complications were reported in 4.1% (n=21) of patients.

**Table 2:** Different surgical procedures in children with cancer

Type of surgery	First surgical procedure, n (%)		Second surgical procedure, n (%)		Third surgical procedure, n (%)	
Biopsy only	255 (50%)		11 (2.2%)		2 (0.4%)	
Partial resection	79 (15.5%)		30 (5.9%)		3 (0.6%)	
Radical resection	170 (33.3%)		54 (10.6%)		5 (1%)	
Metastatectomy	2 (0.4%)		5 (1%)		2 (0.4%)	
Other	1 (0.2%)		0 (0%)		1 (0.2%)	
Total	507 (99.4%)		100 (19.6%)		13 (2.5%)	
Missing values	3 (0.6%)		41 (80.4%)		497 (97.5%)	
Total	510 (100%)		510 (100%)		510 (100%)	

Out of the 92 children who were diagnosed with brain tumors, 86 children underwent surgical procedures. Children with glioma were the group most likely to have a surgical procedure (42/92, 87.5%) (Table 3).

**Table 3:** Surgery versus conservative approach for different types of brain tumors

Brain tumor	Conservative approach, n (%)	Surgical approach, n (%)	Total, n (%)
Atypical rhabdoid teratoid brain tumor	0 (0%)	1 (100%)	1 (100%)
Glioma	6 (12.5%)	42 (87.5%)	48 (100%)
Medulloblastoma	0 (0%)	28 (100%)	28 (100%)
Anaplastic ependymoma	0 (0%)	8 (100%)	8 (100%)
Sialoblastoma	0 (0%)	1 (100%)	1 (100%)
Other tumors of the brain and spine	0 (0%)	6 (100%)	6 (100%)
Total	6 (6.5%)	86 (93.5%)	92 (100%)

Radiation therapy was reported in 13.3% of patients (n=133/1,009) and was well tolerated in 82% of patients (n=109), while 18.1% (n=24/133) developed post-radiotherapy complications. The type of radiotherapy they received was either 3D (n=15/133, 11.3%), conformal (n=95/133, 71.4%), intensity-modulated radiation therapy (n=14/133, 10.5%), or proton therapy (n=9/133, 6.8%). Radiotherapy was used as radical (n=60/133, 45%), palliative (n=11/133, 8.3%), or adjuvant (n=62/133, 46%) treatment. Only five leukemia patients received radiotherapy. Table 4 presents the types of radiation received by children with solid cancers.

**Table 4:** Type of radiation received by children with solid cancers IMRT: intensity-modulated radiation therapy

Brain tumor		3D	Conformal	IMRT	Proton	Total
Glioma	N	1	15	1	1	18
	%	5.60%	83.30%	5.60%	5.60%	100%
Medulloblastoma	N	0	19	1	2	22
	%	0%	86.40%	4.50%	9.10%	100%
Anaplastic ependymoma	N	1	5	0	1	7
	%	14.30%	71.40%	0%	14.30%	100%
Sialoblastoma	N	0	1	0	0	1
	%	0%	100%	0%	0%	100%
Other tumors of the brain and spine	N	1	0	0	0	1
	%	100%	0%	0%	0%	100%
Total	N	3	40	2	4	49
	%	6.10%	81.60%	4.10%	8.20%	100%

The majority of children received chemotherapy (n=891/1,009, 88.3%). The chemotherapy protocols received were either the British protocol (n=426/891, 42%) or the International Society of Pediatric Oncology (SIOP) protocol (n=315/891, 31%). Information was missing regarding the chemotherapy protocol used in 118 children (Table 5). Out of 92 children who had brain tumors, 57 children received chemotherapy (Table 6).

**Table 5:** Different chemotherapy protocols received by children in our study SIOP: International Society of Pediatric Oncology; UK-MRC: United Kingdom-Medical Research Council; FRALLE: French Acute Lymphoblastic Leukemia; COG: Children’s Oncology Group; BFM: Berlin-Frankfurt-Munich

Name of the chemotherapy protocol	Number of patients	Percentage
SIOP	318	31.50%
UK-MRC	426	42.20%
FRALLE	15	1.50%
COG	71	7%
BFM	15	1.50%
Undefined/unspecified	26	2.60%
Other	20	20%
Total	891	88.30%
Missing	118	11.70%
Total population	1,009	100%

**Table 6:** Number of patients who received chemotherapy for different tumors

Brain tumor		No chemotherapy	Chemotherapy	Total
Atypical rhabdoid teratoid brain tumor	N	0	1	1
	%	0%	100%	100%
Glioma	N	25	23	48
	%	52.10%	47.90%	100%
Medulloblastoma	N	2	26	28
	%	7.10%	92.90%	100%
Anaplastic ependymoma	N	4	4	8
	%	50%	50%	100%
Sialoblastoma	N	0	1	1
	%	0%	100%	100%
Other tumors of the brain and spine	N	4	2	6
	%	66.70%	33.30%	100%
Total	N	35	57	92
	%	38%	62%	100%

Almost one-third of the patients developed toxicities related to chemotherapy (n=317, 35%), while 64.4% (n=574) did not report any toxicity; 12% developed late complications in one or more systems related to therapy, and 60% (n=540/891) reported chemotherapy-related complications. Constitutional symptoms were reported in 13.1% of the patients (n=117) such as loss of appetite, weight loss, and general fatigue. The most common early side effects were febrile neutropenia or infections, which were found in 44.1% of the patients (n=393). A detailed presentation of the early complications is given in Table 7.

**Table 7:** Early side effects of chemotherapy in our study population F&FN: fever and febrile neutropenia; BM/BLD: bone marrow/blood; GI: gastrointestinal

	Infection/F&FN	Allergic/immune-related	BM/BLD	GI	Metabolic	Ophthalmic	Skin-related	Pain	Renal
Yes	393 (44.1%)	95 (10.7%)	202 (22.7%)	104 (11.7%)	106 (11.9%)	12 (1.3%)	23 (2.6%)	27 (3%)	14 (1.5%)
No	498 (55.9%)	798 (89.3%)	689 (77.3%)	787 (88.13%)	783 (88%)	879 (98.7%)	868 (97.4%)	864 (97%)	877 (98%)

Late treatment complications were reported in 12.8% of patients (n=129) (Table 8). Late treatment-related complications of the CNS were most often neurological (sensory or motor complications) (n=51, 39.5%), followed by neurocognitive or psycho-social complications.

**Table 8:** Late side effects of chemotherapy in our study population

Type of complication	Number of patients	Percentage
Cardiovascular system	9	0.9%
Central nervous system	36	3.6%
Digestive system	4	0.4%
Endocrine system	25	2.5%
Immune system	3	0.3%
Musculoskeletal	21	2.1%
Reproductive	2	0.2%
Respiratory	2	0.2%
Special senses	22	2.2%
Urinary system	5	0.5%
Total	129	12.8%
Missing	880	87.2%
Total	1,009	100%

Disease status and survival

Post induction or treatment, the majority of patients went into remission (n=790, 78.3%), while some had partial remission (n=35, 3.6%); some developed progressive disease (n=37, 3.7%) or relapsed (n=56, 5.6%). Disease status was followed up at six months, 18 months, and 24 months (Table 9). The majority of children who were lost to follow-up at six months were Kuwaitis (Table 10); 11% of the children were lost to follow-up; they were mainly of preschool age (zero to four years) followed by children of primary school age (five to nine years).

**Table 9:** Disease status of children with cancer post induction or treatment and at follow-up

Status	Post induction		6 months		12 months		18 months		24 months	
	N	%	N	%	N	%	N	%	N	%
Complete remission	790	78.3%	793	78.6%	761	75.4%	703	69.7%	665	65%
Partial remission	35	3.6%	29	2.9%	23	2.3%	18	1.8%	14	1.4%
Progressive disease	37	3.7%	36	3.6%	16	1.6%	11	1.1%	8	0.8%
Relapse	56	5.6%	22	2.2%	22	2.2%	18	1.8%	19	1.9%
Missing data	90	8.9%	116	11.5%	163	16.2%	226	22.4%	266	26.4%

**Table 10:** Nationalities of children with cancer who were lost to follow-up GCC: Gulf Cooperation Council

Patient nationality	Number of patients	Percentage
Kuwaiti	68	58.60%
Non-defined	7	6%
Saudi Arabian	4	3.40%
Egyptian	9	7.70%
Syrian	6	5.10%
Lebanese	1	0.80%
Other GCC countries	1	0.80%
India	7	6%
Bangladesh	1	0.86%
Iran	1	0.86%
Iraq	2	1.70%
Others	6	5.10%
Missing data	3	2.50%
Total	116	100%

**Figure 2:**
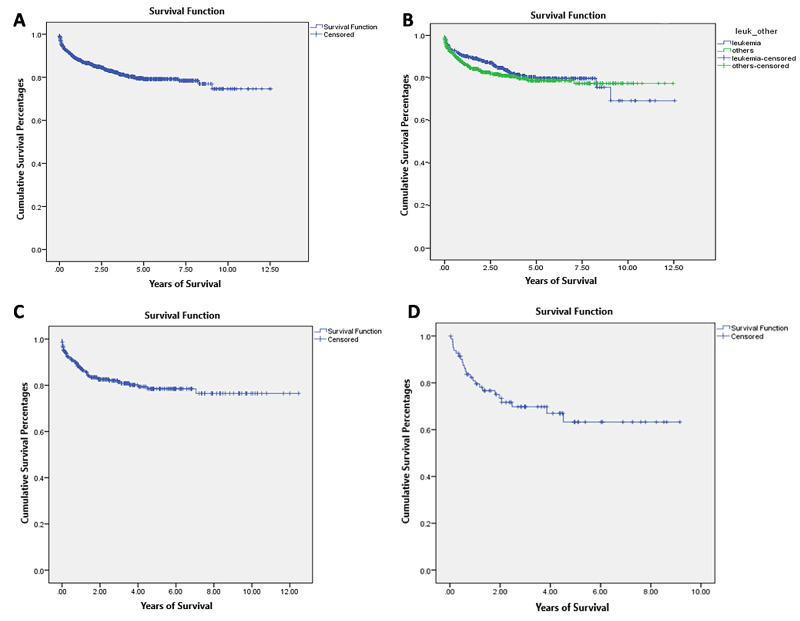
Survival curves of childhood cancer patients in Kuwait A) overall survival rate of children with cancer in Kuwait; B) overall survival rate of children with leukemia in comparison with other cancers in Kuwait; C) overall survival of children with cancers excluding leukemia in Kuwait; D) overall survival rate of children with brain cancer in Kuwait

The overall survival graph shows the survival of children with cancer in Kuwait to be around 80% (Figure 2A). The overall survival rate for leukemia in comparison with other cancers was around 70%, and the overall survival rate for other cancers was around 80% (Figure 2B). The overall five-year survival rate of cancers excluding leukemia was around 80% (Figure 2C), and the 10-year survival rate was almost the same.

Most childhood brain tumors were low-grade gliomas and medulloblastomas. The overall five-year survival rate of brain tumors in children in Kuwait was 60%. This survival curve remains at a plateau at 10 years (Figure 2D). However, this survival curve does not necessarily reflect the scope of brain tumor disease in Kuwait, as some cases skip the registration process at our center.

Major problems related to disease and treatment

Major complications occurred in 9% of patients either due to disease itself or its management. These problems were as follows: relapse, fatal sepsis, obesity, secondary cancer, or neurological sequelae. Relapse occurred in 53 children either within two years, five years, or even after five years of treatment. Fourteen children were lost to fatal infections, and four children reported secondary cancer (Table 11), i.e., 1:252 developed secondary cancer. Some children required second-line treatment (n=128, 12.7%). Bone marrow transplant was the only treatment option in 6.3% of children (n=64) and some children (2.8%, n=28) had persistent or aggressive diseases or developed a debilitative state and required palliative care. Salvage treatment was the main option in 4.8% (n=48/128) of children who required second-line treatment, while targeted treatment was the treatment option for 0.8% (n=8/128) of children. The majority of children were alive and well when last seen. Unfortunately, 10% passed away, 11% were lost to follow-up, and 3% were left with a permanent disability (Table 12).

**Table 11:** Major complications in children with cancer in Kuwait CNS: central nervous system

Major complication	Number of patients	Percentage
Obesity	3	0.30%
Severe weight loss	2	0.20%
Major neurological sequelae	15	1.50%
Hematological relapse after 2 years	37	3.70%
CNS relapse after 2 years	2	0.20%
Relapse within 5 years of stopping treatment	10	1%
Relapse after 5 years of stopping treatment	3	0.30%
Fatal sepsis	14	1.40%
Secondary cancer	4	0.40%
Total	90	8.90%
Missing values	919	91.10%
Total population	1,009	100%

**Table 12:** Status at last contact of children with cancer in Kuwait

Status at last contact	Number of patients	Percentage
Living and well	770	76.30%
Deceased	95	9.40%
Living and debilitated	31	3.10%
Lost to follow-up	113	11.20%
Total	1,009	100%

## Discussion

Cancer is a major health problem in terms of morbidity and mortality worldwide and especially in developing countries [7]. The incidence rates of childhood cancer have been increasing, and so have the survival rates [8]. Although there has been an improvement in the five-year survival rates, childhood cancer is the second leading cause of death after accidents in developed countries [9]. Moreover, 30% of childhood cancer deaths are due to hematological malignancies [10].

There are ethnic, racial, and geographic variations in the incidence of childhood cancer and survival worldwide [11]. In our study, almost 60% of patients were Kuwaitis, and the rest were non-Kuwaitis of different nationalities. The incidence rate we calculated was similar to the incidence rate in other countries [12,13]. Furthermore, incidence rates were higher in males compared to females, which is consistent with all international statistics [14].

With regard to cancer risk factors, the most common genetic disease reported was Down syndrome [15]. This may be explained by the fact that Kuwaiti women choose to have children throughout their fertile years, which may increase the incidence of Down syndrome in their later pregnancies. Additionally, consanguineous marriages are prevalent among the Kuwaiti population, which poses an added risk [16]. Past medical history was not common, and the most commonly reported disease was a cardiac disease. This can be explained by the recent studies linking cancer with cardiac diseases, as congenital cardiac diseases have been associated with the development of cancer [17]. Our findings of the prevalence of a positive family history of cancer were similar to other studies, and the majority of the positive family history was related to members of patients' extended family [18,19].

The majority of parents had a higher education degree, which does not match the general description of the population in Kuwait, where the majority have intermediate education only [20]. We hypothesize that parents who have intermediate education are mostly expats and do not have the means to support families in Kuwait, and so their children do not contribute to Kuwait’s childhood cancer statistics [2]. The majority of families in this study were middle- or high-income families, and members were well-educated, and this is consistent with the studies that have associated the parents' education and socioeconomic status with long-term survival, especially for cancers that require long-term treatment [21].

According to a Global Cancer Observatory (GLOBOCAN) estimation of cancer rates in 2018, leukemia was the most common childhood malignancy worldwide, followed by malignancies of the brain and nervous system, non-Hodgkin lymphomas, renal tumors, and Hodgkin lymphomas. However, the rank and outcome of the most common cancer types varied among countries [22]. Our results were almost similar to other registries in the region [23].

The incidence of leukemia we calculated was higher than the general international incidence [24]. This is probably due to the fact that we could not retrieve the files for the children with oncology diseases and only retrieved the files for leukemia patients for the year 2004. Some files from 2004 and the following five years were not brought to our center and hence were lost from the registry. The distribution and five-year survival rates of different types of leukemia were in line with international statistics [8,25]. The overall survival for leukemia has improved; the five-year survival was around 80%, and the overall 10-year survival for leukemia was 70%.

The second most prevalent tumor in children in Kuwait was lymphoma, which is not the case elsewhere where brain tumors have a higher incidence than lymphoma. One reason could be the overlap of lymphomas and leukemia with regard to the clinical presentation and course of the disease, as we have recorded a few children with lymphoma who presented with bone marrow infiltration. Many children were diagnosed in the pediatric neurosurgery department and had surgeries, or traveled abroad seeking specialized treatment for complicated cases, or died without showing up at our center. Similar problems have been documented in other studies [26]. Nevertheless, more research is needed to explain the increased incidence of lymphoma. The survival rate of lymphoma was high, which is in keeping with international statistics, and this reflects the improvements in the treatment methods of Hodgkin and non-Hodgkin lymphoma.

Toxicities related to chemotherapy developed in around a third of the patients. One of the least reported complications was pain (3%), it could be attributed to underreporting or the sound pain management protocol that was developed in 2011. Endocrine problems were rarely reported, although steroid-induced hyperglycemia is frequently witnessed in clinical practice. One explanation is that these complications were mistakenly counted with metabolic/laboratory complications. Late treatment complications were also reported in a small number of patients, which contrasts with other studies [27]. Hence, we believe they were underreported.

The majority of children went into remission after treatment, and this is consistent with international statistics [28]. The number of children in remission at presentation was higher than at 24 months as some children were lost to follow-up as they had either traveled abroad for further treatment or had relapsed and were referred to another specialty for further treatment. The number of patients who developed secondary cancer in our study was higher than the number documented in other studies [29]. This mandates creating an algorithm to identify children at risk of secondary cancer to avoid adverse outcomes.

The survival rate for all children with cancer in Kuwait was high, which is in line with international statistics, and so was the incidence in comparison to survival rates [1,30]. This may be a good reflection of the services provided, but it does not address the survival rates of different cancers. Our study population had a heterogeneous genetic make-up, and this might have affected cancer prevalence and management as survival differs among different types of cancer. In addition, many of our patients with the same disease received different treatment protocols according to the treatment center, and this might have influenced our results.

This project has transformed the way we gather information in our center. We have worked on standardizing the data collection process on the registration of new patients to prevent the obstacles we faced in our study.

Limitations

The data collection process for the childhood cancer registry was not comprehensive. The dated manual system and the transfer of patients between hospitals prior to 2010 made it impossible to get all the required data from files from other hospitals. Many files were excluded from the study because they lacked essential information, and almost 11% of enrolled children were lost to follow-up, which may have affected the survival curve.

## Conclusions

Based on our findings, the trends in childhood cancer statistics in Kuwait share some similarities with international statistics. However, the incidence of leukemia was higher than the international rates, and the second most common cancer was lymphoma, rather than brain tumors. Survival rates were as high as in other countries, which reflects the good standard of care in Kuwait. A hospital‐based cancer registry and active data management program are required to explore the difference in incidence rates in Kuwait compared to other countries and measure the effectiveness of specific interventions to improve the quality of care. Funding registries such as ours would help in the improvement of childhood survival through continuous assessment and auditing of the services provided.
